# Determining the efficacy of guppies and pyriproxyfen (Sumilarv® 2MR) combined with community engagement on dengue vectors in Cambodia: study protocol for a randomized controlled trial

**DOI:** 10.1186/s13063-017-2105-2

**Published:** 2017-08-04

**Authors:** John Hustedt, Dyna Doum, Vanney Keo, Sokha Ly, BunLeng Sam, Vibol Chan, Neal Alexander, John Bradley, Didot Budi Prasetyo, Agus Rachmat, Shafique Muhammad, Sergio Lopes, Rithea Leang, Jeffrey Hii

**Affiliations:** 1 Malaria Consortium, House #91, St. 95, Boeung Trabek, Chamkar Morn, PO Box 2116, Phnom Penh, 12305 Cambodia; 2 London School of Hygiene and Tropical Medicine, Keppel Street, London, WC1E 7HT UK; 3 Cambodian National Dengue Control Program, #477 Betong Street.(Corner St.92), Village Trapangsvay, Phnom Penh, Cambodia; 4 World Health Organization, No. 177-179 corner Streets Pasteur (51) and 254; Sankat Chak Tomouk Khan Daun Penh, Phnom Penh, Cambodia; 5 US Naval Medical Research Unit-2, #2, St. 289, Boeung Kok 2 commune, Toul Kork district, 289 Samdach Penn Nouth, Phnom Penh, 1225, Cambodia

**Keywords:** Dengue, Guppy, Pyriproxyfen, Community engagement, Vector control, Cambodia

## Abstract

**Background:**

Evidence on the effectiveness of low-cost, sustainable, biological vector-control tools for the *Aedes* mosquitoes is limited. Therefore, the purpose of this trial is to estimate the impact of guppy fish (guppies), in combination with the use of the larvicide pyriproxyfen (Sumilarv® 2MR), and Communication for Behavioral Impact (COMBI) activities to reduce entomological indices in Cambodia.

**Methods/design:**

In this cluster randomized controlled, superiority trial, 30 clusters comprising one or more villages each (with approximately 170 households) will be allocated, in a 1:1:1 ratio, to receive either (1) three interventions (guppies, Sumilarv® 2MR, and COMBI activities), (2) two interventions (guppies and COMBI activities), or (3) control (standard vector control). Households will be invited to participate, and entomology surveys among 40 randomly selected households per cluster will be carried out quarterly. The primary outcome will be the population density of adult female *Aedes* mosquitoes (i.e., number per house) trapped using adult resting collections. Secondary outcome measures will include the House Index, Container Index, Breteau Index, Pupae Per House, Pupae Per Person, mosquito infection rate, guppy fish coverage, Sumilarv® 2MR coverage, and percentage of respondents with knowledge about *Aedes* mosquitoes causing dengue. In the primary analysis, adult female *Aedes* density and mosquito infection rates will be aggregated over follow-up time points to give a single rate per cluster. This will be analyzed by negative binomial regression, yielding density ratios.

**Discussion:**

This trial is expected to provide robust estimates of the intervention effect. A rigorous evaluation of these vector-control interventions is vital to developing an evidence-based dengue control strategy and to help direct government resources.

**Trial registration:**

Current Controlled Trials, ID: ISRCTN85307778. Registered on 25 October 2015.

**Electronic supplementary material:**

The online version of this article (doi:10.1186/s13063-017-2105-2) contains supplementary material, which is available to authorized users.

## Background

Dengue is one of the most rapidly spreading mosquito-borne viral diseases in the world, and is caused by bites of infected *Aedes* mosquitoes, principally *Aedes aegypti* [1]. Dengue infection is a systemic and dynamic disease with a wide clinical spectrum that includes both severe and non-severe manifestations and in some cases can lead to death [1]. With an estimated 3.6 billion people in 124 countries at risk of contracting the disease [2] and 390 million dengue infections occurring each year (of which 96 million are clinically apparent) [3], the dengue virus has become a leading cause of illness in the tropics and subtropics [4]. Asia records 70% of the global disease burden due to dengue [3] and Cambodia has one of the highest per-capita incidence rates in the region [5].

Identified in Cambodia in 1963 [6], a total of 194,726 dengue cases were reported to the National Dengue Control Program (NDCP) between 1980 and 2008 [7]. Between 2003 and 2008, annual dengue incidence ranged between 0.7 and 3.0 per 1000 persons, the cost to society estimated at between US$3,327,284 and US$14,429,513 [8]. Since most of this cost falls onto the family, it is estimated that 67% of affected households (HHs) fall into debt to pay for medical bills [9]. However, it is likely that the real number of cases and cost to society is much greater, with some studies suggesting the real case numbers are between 3.9 and 29.0 times higher than those of the National Dengue Surveillance System [10, 11].

Although a number of promising vaccine candidates are in preclinical and clinical development [12], and methods of genetic control of mosquitoes are being developed [13–15], they are years from operational roll-out in Cambodia and are unlikely to provide universal protection. Without a cure or vaccine the most appropriate dengue control measures are vector control and the avoidance of mosquito bites. Several vector-control methods have been studied in Cambodia including chemical and biological substances (temephos, pyriproxyfen (PPF), and *Bacillus thuringiensis israelensis* (*Bti*)) [16–19], jar covers [20], and distribution of larvivorous copepods and fish [21–23].

### Past research

*Ae. aegypti* is highly anthropophilic (preference for human beings), endophilic (resting indoors), and endophagic (preference for feeding indoors) [19]. This partially explains why previous studies showed that household water storage jars contained over 80% of *Ae. aegypti* larvae in Cambodia, and why these jars became the main target for dengue vector-control activities [20]. Since the early 1990s, the NDCP has used the larvicide temephos (distributed with brand name Abate®) to target large (200–400 L) household water containers as the primary means of vector control [19]. This has continued despite susceptibility tests in 2001 showing resistance of *Ae.* a*egypti* in urban Phnom Penh and incipient resistance in a rural province in Cambodia [24]. An evaluation of the effectiveness of temephos control programs to control larvae in 2007 concluded that control strategies emphasizing the use of temephos should be reconsidered [19]. Although temephos was only distributed during the rainy season, there were still containers found to be positive for immature *Aedes* during the dry season; and the program ignored discarded containers – which had twice the number of larvae as water storage containers. Khun and Manderson (2007) concluded that “continued reliance on temephos creates financial and technical problems, while its inappropriate distribution raises the possibility of larvicide resistance [19].”

Following the success of *Mesocyclops* (a genus of copepod crustaceans) programs in locally eliminating *Aedes* mosquitoes in Vietnam [25–27], the Cambodian NDCP implemented a 2-year *Mesocyclops* project in Kratie province from 2002 to 2004, searching for an alternative vector-control option [23]. Initial results showed a reduction in the *Aedes* population in the intervention area, but by the end of the project larval densities in the intervention area had increased by 62% from baseline. This apparently lower effectiveness in Cambodia may be because *Mesocyclops* from the local water sources had various parasites, and colonizing them parasite-free requires special training beyond what is possible in most rural Cambodian villages. The environment could have played a role as Northern Vietnam (where programs were most successful) has four distinct seasons and has different flora and fauna. Additionally, many people did not accept *Mesocyclops* to the same extent as other interventions that were provided by the NDCP such as temephos (personal communication, To Setha, 2015).

The search for other vector-control options continued with an evaluation of *Bacillus thuringiensis israelensis* (*Bti*), a Gram-positive, soil-dwelling bacterium used as a biological control agent [18]. The evaluation of *Bti* in Phnom Penh showed positive results with significant reductions in the number of pupae for at least 2 and 2.5 months in containers with river and well-water, respectively [18]. More extensive usage and evaluation of *Bti* by the Cambodian government are currently occurring in Kandal and Kampong Thom Provinces (personal communication, Bunleng Sam, 2015).

Jar covers with long-lasting insecticidal netting (LN) treated with deltamethrin were found to have significantly fewer pupae per house, a three-fold decline in *Ae. aegypti* adult females per house and adult mosquito survival [20]. However, the magnitude of the reduction diminished over time due to a gradual reduction of insecticidal effect of the jar covers and a residual deltamethrin life of 22 weeks [20]. Interestingly, this is less than the average residual life of deltamethrin in treated bed nets [21]. Another cause may have been children not always keeping the jar covering on after extracting water, and using the covers as toys around the house (personal communication to Setha, 2015) as Khun et al. noted in Cambodia before [28]. Improvements in engineering and design to prevent entry and egress of mosquitoes, especially when the container is used, and an increase the insecticidal effectiveness may be needed for jar covers to be cost-effective public health interventions [20].

The use of a larvivorous guppy fish (guppies) (*Poecilia reticulata*) was evaluated in 14 Cambodian villages [21], and subsequently in a larger study of 28 Cambodian villages [22]. Results from the initial study done from 2006 to 2007 were extremely encouraging with guppies in 56% of eligible containers, and a 79% reduction in *Aedes* infestation compared to the control. Guppy fish are not able to colonize all potential *Aedes* breeding sites, especially those which are polluted or with volume of less than 50 L (personal communication to Setha, 2015). However, despite not having guppies, the smaller or discarded containers in the intervention villages had 51% less infestation than those in the control, suggesting a community-wide protective effect [21]. This could partly be due to a spillover effect from treatment villages as no results of guppy coverage were reported in the paper. These results led the World Health Organization (WHO) and the Asian Development Bank (ADB) to fund a larger scale-up in 2010–2011 which included Communication for Behavioral Impact (COMBI) activities. Results showed 88% guppy fish coverage in eligible water containers and a Container Index (CI), i.e., proportion of surveyed containers containing *Ae. aegypti* larvae/pupae and indoor resting adult females of near 0 (while the control had a CI of 30) at the end of the project [22]. Similarly encouraging results were found in Laos as a part of the same project. However, there were additional miscellaneous breeding sites including containers too small for guppy survival. Additional tools beyond larvivorous fish are required to target these varied, hard-to-reach and cryptic breeding containers or sites.

One such alternative that has been evaluated in Cambodia is pyriproxyfen (PPF) [16, 17]. PPF is a juvenile hormone analog that interferes with the metamorphosis of juvenile *Aedes* mosquitoes, preventing their development into adults [29]. The results of the first study in 2003 were so promising – at higher doses, inhibition of adult emergence (IE) greater than 87% for 6 months – that a larger second study was designed [16]. This showed that a novel 5% controlled-release formation led to IE above 90% for 20 weeks, and above 80% for 34 weeks [17]. A slow-release PPF matrix release formulation (Sumilarv® 2MR) has since been developed and is suitable for containers uninhabitable by guppy fish. The added benefit of this product is that it only requires one distribution every 6 months (the entirety of the rainy season) and cuts down on operational costs as compared to temephos or *Bti* which have a residual efficacy of 2–3 months [18, 30].

The effective implementation of Integrated Vector Management requires mobilization and coordination of the resources needed to achieve and sustain behavior changes among populations at risk of dengue [31]. The COMBI strategy provides a social mobilization and communication approach that connects knowledge and behavior, addresses the cost and value of engaging in healthy behaviors, recognizes the gradual stages of behavior change, and creates a supportive environment for behavior change [32]. The challenge for vector control is how community participation can be integrated into vector breeding-source reduction efforts [22]. Community health workers (CHWs) are a vital part of successful COMBI communication and social mobilization activities. A recent review of 22 studies found that educational messages embedded in a community-based vector-control approach were effective at reducing *Ae. aegypti* measured through entomological indices [33]. A separate systematic review found that community-based control strategies in addition or together with biological and chemical vector-control tools reduced classical *Aedes* larval indices in five of six field trials [34]. Two cluster randomized trials published after the reviews showed that a community empowerment strategy embedded in a routine dengue vector-control program drastically reduced entomological indices [35, 36]. These past studies show the importance of having a strong community-based COMBI strategy, and the important contribution that it can add when integrated into the vector management strategy.

### Need for a trial

Although there is evidence suggesting that the use of guppy fish can be beneficial in dengue vector control, recent reviews show that there has never been a cluster randomized trial to evaluate their effectiveness to reduce mosquito indices [37]. Although some studies have evaluated the use of community-based communication programs for dengue control, a recent review found that none had assessed their costs [34]. This trial has the potential to inform the strategic application of community-based distribution of PPF and larvivorous fish in an outbreak, during inter-epidemic periods or for broad-scale application. This trial will also be the first to our knowledge to evaluate the wide-scale use of the new Sumilarv® 2MR product in the field (personal communication, John Lucas, 2015). Although guppies, PPF, and COMBI activities have all been evaluated, some of these evaluations were methodologically flawed. Furthermore, they have never been tested in combination. Therefore, our study is intended to fill the knowledge gaps identified above.

### Hypothesis

This trial aims to demonstrate community effectiveness of guppies, PPF, and COMBI activities. The main hypotheses are:

1. Use of guppies, Sumilarv® 2MR, and COMBI activities will reduce numbers of *Aedes* mosquitoes, and their infection rates, more than guppies and COMBI alone, or usual Ministry of Health activities (including larval control and information and education material dissemination during outbreaks) as assessed through entomology surveys
2. COMBI activities will improve the community’s knowledge, attitudes, and behavior around water use and vector-borne disease prevention (such as burning or burying discarded containers, cleaning the environment around the house, and sleeping under a bed net) as assessed through baseline/endline surveys and Focus Group Discussions (FGDs)
3. Guppies and PPF will be acceptable among the target villages as assessed by an endline survey and FGDs

The study is designed as a cluster randomized controlled, superiority trial with three parallel arms.

## Methods/design

This protocol is reported following the Standard Protocol Items: Recommendations for Interventional Trials (SPIRIT) criteria [38]. For the completed SPIRIT Checklist see Additional file 1.

### Study setting

**Fig. 1:**
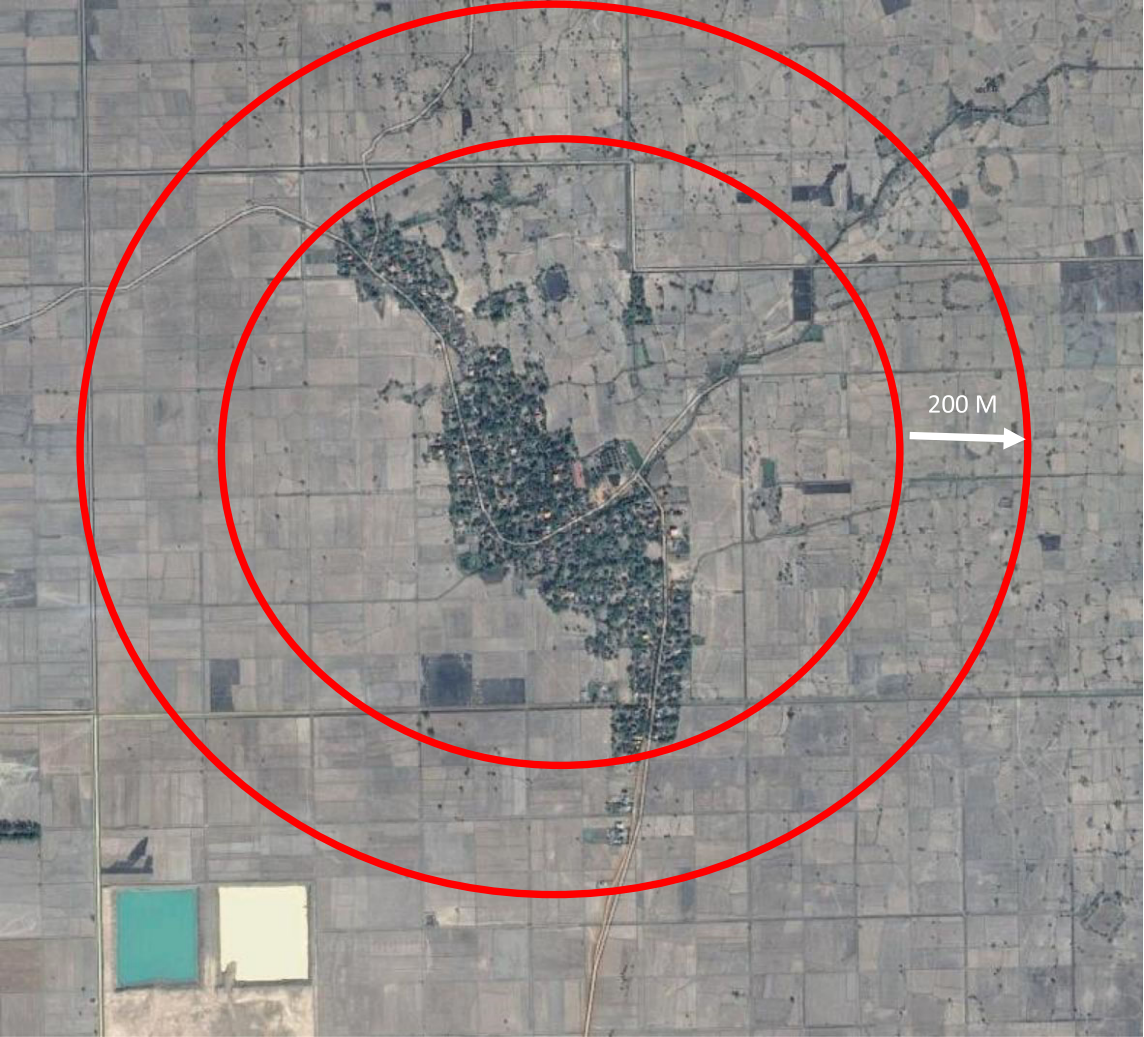
Example of a 200-m boundary around selected clusters

The study has 30 clusters in two operational districts (ODs) (one OD includes the jurisdiction of 10 health centers (HC) or roughly 100,000–200,000 individuals) within Kampong Cham province. Each cluster has on average 200 HHs or 1000 individuals. The rainy season runs from April to November, and the peak dengue season is from May to July. Kampong Cham was selected as it has one of the highest dengue incidence rates of 1.6 cases per 1000 people in Cambodia and the environmental characteristics are similar to most dengue-endemic areas of Cambodia (personal communication, Hai Ra, 2016). The clusters (containing one or more villages) were selected based on availability of entomological surveillance data from previous surveys. To avoid spillover effects, clusters had to be at least 200 m from the nearest HH outside the cluster since *Ae. aegypti* in this region have an average flight range of 50–100 m [39] (see Fig. 1).

#### Eligibility criteria

Every house within the cluster boundaries will be invited to participate in the trial.

#### Interventions

**Fig. 2:**
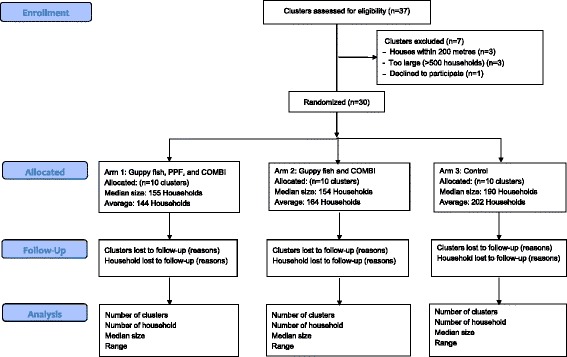
Flow chart of cluster selection. Selection of clusters in Kampong Cham, Cambodia

**Fig. 3:**
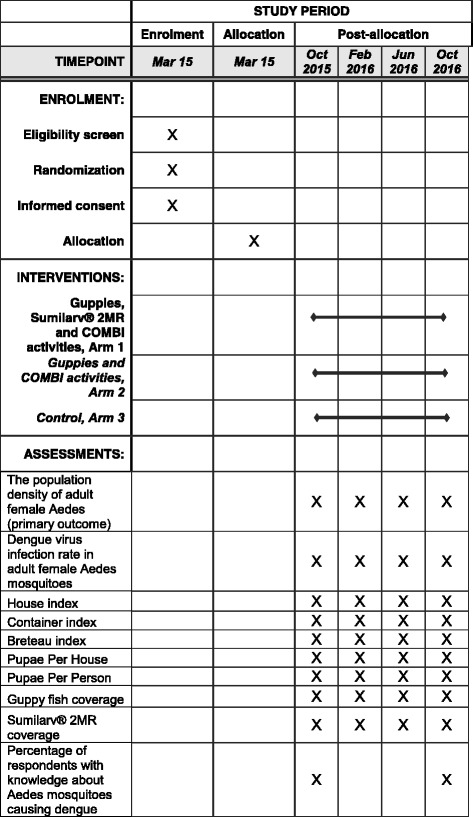
Standard Protocol Items: Recommendations for Interventional Trials (SPIRIT) with schedule of enrollment, interventions, and assessments

Selected villages will be randomized into one of three study arms (see Table 1). Study arm 1 receives all three interventions, while study arm 2 receives only guppies and COMBI activities, and arm 3 will receive only the standard vector-control activities from the Ministry of Health. The total trial period for the interventions will be 11 months (see Figs. 2 and 3).

**Table 1:** Interventions randomized to each study arm

Intervention	Arm 1	Arm 2	Arm 3
Guppy fish in key containers (>50 L)	X	X	
Communication for Behavior Impact (COMBI) activities	X	X	
Direct pyriproxyfen (PPF) application (Sumilarv® 2MR) in smaller containers (10–50 L)	X

Study arm 1 was chosen to evaluate the effectiveness of all three interventions in combination. Application of any insecticide can be expensive when taking into account procurement and operational costs. Arm 2 was selected to evaluate the effectiveness of less expensive interventions (guppies and COMBI), although all strategies are expected to be less expensive than current strategies. As COMBI-related activities have been shown to have a significant impact on the coverage of interventions in Cambodia and elsewhere [21, 22, 34] a guppy-only arm was not included. Therefore, the trial will not give separate estimates of the effects of guppies and COMBI. Larvicide-only arms were not included because larvivorous fish are more sustainable and cost-effective than larviciding [16, 17, 21, 22, 40, 41], and if larviciding was found to be equally effective, guppies would be recommended in terms of cost and acceptability.

### Guppies

In rural Cambodia, more than 80% of *Aedes* mosquito breeding is detected in key containers, such as large water jars, cement tanks and other large containers, used for the storage and collection of water for human and animal consumption and washing [20]. To target these containers, two guppy fish (*Poecilia reticulata*) will be placed into each water container with a volume greater than 50 L in intervention villages (arms 1 and 2). This is based on larval consumption of guppies determined by Seng et al. [21] and past experiences using guppies in vector control in Cambodia [22]. The guppy fish will be distributed after the baseline activities through a local community network managed by provincial government authorities. Guppy banks will be set up at the corresponding health centers and consist of twenty 500-L jars. Guppy banks will be colonized and can provide fish at any time to CHWs in implementation villages. One CHW will be assigned to monitor 30 HHs each month. They will each have two 500-L jars which they can colonize with guppies to provide for their assigned HHs. When CHWs need more guppies they can return to the guppy bank for them. Each month CHWs will conduct visual checks and ensure that all their assigned HHs have guppies in all large containers, and replace them if necessary. Adult guppies are on average 1.5–3.5 cm long (males) or 3–6 cm long (females) [42].

### Pyriproxyfen matrix release (Sumilarv® 2MR)

**Fig. 4:**
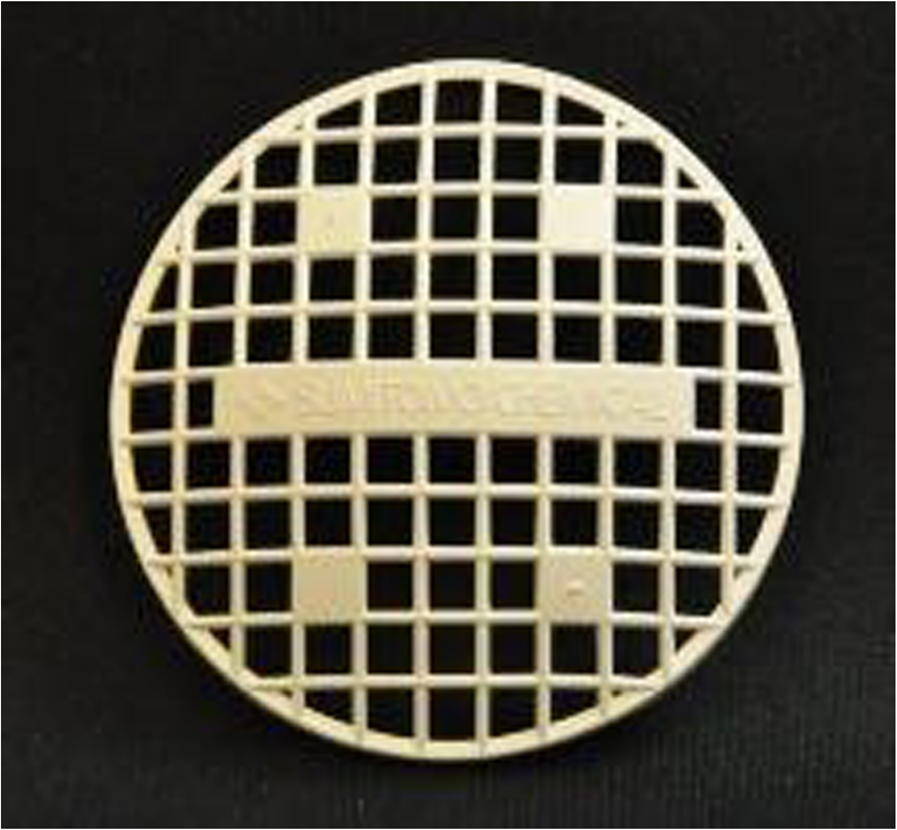
Sumilarv® 2MR disk (5-cm radius)

Each device or disk (See Fig. 4) is designed to provide coverage for 40 L of water. It is also possible to cut disks into smaller sizes for smaller-sized containers (see Table 2).

**Table 2:** Dosage application rate of Sumilarv® 2MR disks (target dosage is one disk per 40 L)

Container capacity, L	Number of 2MR disks	Target ppb
10	1/5	27
20	½	27
30	2/3	27
40	1	27
50	1	27

*ppb* parts per billion

PPF devices will be distributed after the baseline survey in the same manner as described above, and replaced after 6 months. Additional devices will be left at the health center for CHWs to distribute during their monthly monitoring visit if some have been lost or need to be replaced.

Although there have not been any studies evaluating the safety of PPF in humans, toxicity to fish is induced at 450 ppb, which is approximately 45 times greater than the target ppb (10) of Sumilarv® 2MR [43]. The LD50 in rats is 5,000,000 ppb, or 500,000 times the target concentration [44]. These data suggest a very favorable mammalian toxicity profile, and extremely low risk for humans using this product.

### Communication for behavioral impact activities

An initial rapid assessment consisting of FGDs and In-depth Interviews (IDIs) regarding knowledge, attitudes, and behaviors of community members was completed. The results were used in a message and material development workshop held with key community and district stakeholders. During this meeting the community helped to develop behavior change communication materials and come up with key messages. The results were used to understand the common social gathering locations for health education sessions, culturally appropriate channels of communication, and to create communication materials: flip charts to guide CHW education sessions, posters and banners for display in the villages, songs, and CHW materials such as hats, t-shirts, bags, and rain coats.

A 2-day training will be given to CHWs on communication and facilitation skills, following which they will take the lead role in conducting health education sessions in their community. A monthly meeting will also be conducted with CHWs to assess progress, address issues and challenges, and provide them with continuous training to develop their confidence and skills. The health education sessions will occur twice per month and will be participatory, as Khun and Manderson [28] found that health education sessions, where participants actively identify breeding sites and practice positive behaviors, can be more effective and less costly than the didactic classroom-based sessions. In addition to health education sessions, we will use locally available media, such as loud speakers fixed to local transport, to play songs, street theatre performances, and role playing to reinforce the messages.

#### Adherence

In order to improve adherence to the intervention protocols, CHWs will perform monthly monitoring checks on each HH within the intervention arms. The presence or absence of guppy fish and PPF Sumilarv® 2MR in each container within the HH will be recorded along with any replacements the CHWs provide. The entomology surveys will also record the presence or absence of each intervention in containers (including those used for domestic and non-domestic use) within the surveyed HHs. Project staff will also randomly visit CHWs and intervention HHs to confirm the reliability of data provided.

#### Primary outcome measures

The primary outcome measure is the population density (i.e., number of mosquitoes per unit of time spent aspirating) of adult female *Aedes* trapped using adult resting collections.

#### Secondary outcome measures

The secondary outcomes for the trial include:

1. Dengue virus infection rate in adult female *Aedes* mosquitoes
2. House Index (HI): proportion of houses surveyed positive for *Aedes* larvae and/or pupae in any water container
3. Container Index (CI): proportion of surveyed containers containing *Aedes* larvae and/or pupae
4. Breteau Index (BI): number of containers positive for *Aedes* larvae and/or pupae per 100 houses surveyed
5. Pupae Per House (PPH): number of *Aedes* pupae per household
6. Pupae Per Person (PPP): number of *Aedes* pupae per person
7. Guppy fish coverage: proportion of eligible water containers with at least one guppy fish
8. Sumilarv® 2MR coverage: proportion of eligible water containers with at least one MR disk
9. Percentage of respondents with knowledge about *Aedes* mosquitoes causing dengue

#### Sample size

The guppy fish and PPF interventions will be assessed by an entomology survey. A sample size of 10 clusters per arm and 40 HHs per cluster for the survey was devised using the Hemming and Marsh method [45]. The calculation assumed a mean of 0.1 adult female resting *Aedes* mosquitoes per HH in the intervention arms compared to 0.25 in the control arm for each collection. This assumption was based on the results from the earlier World Health Organization/Asian Development Bank guppy fish project in the same province [22], and to be conservative assumed no impact from the PPF in arm 1. The HHs will be randomly selected at each collection. The intracluster correlation (ICC) was assumed to be 0.01 based on previous studies [46–48]. Additionally, a sensitivity analysis was conducted up to the median value of ICCs for outcome variables (0.03) as found by an analysis conducted by Campbell et al. [49]. Our analysis determined that we would have between 91 to 75% power at ICC values between 0.01 to 0.03. The coefficient of variation in cluster sample size was assumed to be 0.1, and is expected be small as we plan to sample the same number of houses from each cluster. Under these assumptions the study will have 91–75% power to detect a difference at the 5% significance level.

COMBI activities will be evaluated through Knowledge, Attitudes, and Practice (KAP) surveys. A sample size of 10 clusters per arm and 20 HHs per cluster was devised, again using the Hemming and Marsh method [45]. The calculation assumed a 22.5% change in primary outcome indicators from 40 to 62.5% in intervention villages and no change in the control villages over the course of 1 year. Outcome indicators include:

1. Percentage of respondents with knowledge about *Aedes* mosquitoes causing dengue
2. Percentage of HHs that keep guppy fish/PPF in water

This calculation was based on the results from the earlier projects done by Malaria Consortium (MC) in the region, and a recent unpublished MC KAP survey completed in six provinces and 30 villages in 2014. The ICC was assumed to be 0.01, and the coefficient of variation of cluster size assumed to be 0.1. Under these assumptions the study will have 90% power to detect a difference at the 5% significance level.

#### Allocation

Clusters will be randomly assigned with a 1:1:1 allocation through a public randomization process. Village chiefs from all clusters and HC chiefs from all HCs will be invited to a central point along with local and national authorities. Locally, the concept of “lucky draw” is very popular, so this method will be used to randomize clusters. Each cluster representative will choose one paper labeled either arm 1, 2, or 3 from a bowl. The numbers on the papers will be printed and concealed by folding the paper in half four times. Three large, labeled sheets of paper will be put on the wall. As each representative selected their study arm, MC staff will write the cluster names on the corresponding sheet.

#### Data collection methods

Data will be collected at 0, 4, 8, and 12 months post intervention, unless otherwise mentioned. The project will employ the following methods:

### Entomology

A baseline survey was conducted prior to start of interventions. An endline survey will be conducted 1 year after the baseline. Two additional surveys during the dry season (4 months post intervention) and light rain (8 months post intervention – peak dengue season) will also be conducted. The schedule of the surveys took into account data from the previous guppy fish project [22]. The survey methodology was developed following the WHO guidelines for entomological collections [1]. Surveys will include indoor adult resting catches and larvae/pupae collection from water containers. The survey team consisted of experienced government staff who received 3 days’ training before beginning. All tools and materials were pre-tested during training. The same team will be used for each entomology survey. Houses within each cluster were selected using a random-number generator [50] applied to the village list managed by the village head.

The adult resting catch will be completed using a battery-powered, portable aspirator (Camtech, Phnom Penh, Cambodia) for 10 min per house in the bedrooms and living spaces, starting in the bedroom and aspirating up and down the wall (from floor to 1.5 m) around the home in a clockwise manner. The mosquitoes will be kept in a screw-top containers inside a cold box and transported to the provincial laboratory for identification to the species level for *Aedes*, otherwise to genus. All *Aedes* mosquitoes will be sexed. After identification they will be stored in a −20 °C freezer and taken to the United States Naval Medical Research Unit 2 (NAMRU-2) in Phnom Penh for confirmation. All *Aedes* females will be pooled and subjected to flavivirus rRT-PCR screening [51]. Flavivirus-positive pools will be further tested for dengue typing by semi-nested RT-PCR assay targeting the C and pre-M regions within the viral genome [52].

Larvae and pupae collection will be completed using the five-sweep net method [53] for containers larger than 50 L. The size of the net is 20 cm by 33 cm. Surveyors will turn the net in an anti-clockwise manner five times, then wait 1 min and perform one sweep from the bottom. This method can sample around 35% of larvae and 31% of pupae, and the total number estimated by an adjustment factor [53]. For containers of less than 50 L, all the water will be poured through the sweep net. All containers within selected HHs will be inspected. All pupae and 10 larvae per container will be put in a plastic bag, labeled (with date and code), and taken back to the laboratory for identification to the species level for *Aedes*, otherwise to genus. After identification they will be taken to NAMRU-2 in Phnom Penh where entomologists will confirm identification of a random sample of 50% of immature mosquitoes.

Survey teams will also record the number, size, and type of all water containers in the HH The team will complete a rapid assessment tool (Premise Condition Index; PCI) [54] to identify whether the scores can predict HH risk for *Ae. aegypti* infestation. If proven useful as an indicator of risk, PCI could be used to streamline future surveys and program activities and possibly reduce program costs.

### Knowledge, attitudes, and practices

The KAP survey was designed around the results of the FGDs and IDIs to create questions based on the local context and language [55]. The KAP will be pilot tested in a neighboring community, and revised where necessary. Questions are close ended or are categorized by data collectors at the time of response.

KAP surveys will be conducted at the same time as baseline and endline entomology surveys. Only the HH head will be asked to respond. The data will be collected by experienced government staff who will be given 2 days’ training before implementation. Each team will have a supervisor who can monitor data integrity and quality. All paper forms are submitted to the MC supervisor who performs a final check making sure that all questions receive a response and that skip patterns are followed correctly.

### Community health worker monthly monitoring

The coverage of guppy fish and PPF Sumilarv® 2MR will be assessed by ocular inspection of water containers via entomology surveys and the CHW monthly reporting form as described in the adherence section. Coverage is expressed as percentage of containers with guppy fish or Sumilarv® 2MR of the total HHs or containers examined.

### Location

The geographical location of each village within the trial and all HHs in the entomology/KAP surveys will be recorded by a handheld Global Positioning System and plotted using ArcGIS® version 10 (Environmental System Research Institute, Redlands, CA, USA) for spatial analysis and for presentation purposes.

### Climate

General climate data (rainfall, temperature and humidity) will be recorded at one of the intervention health centers using a rain gauge and a Hobo onset data logger (all villages have virtually the same climate). Data from the all United States National Aeronautics and Space Administration (NASA) satellites on climate will also be available including air pressure, air temperature, atmospheric moisture, evaporation, precipitation, and wind [56].

### Data management

Double data entry into EpiData (EpiData Association, Odense, Denmark) is completed by an experienced data-entry company. The process of data cleaning is being handled by MC staff. The original forms are kept in a secure, locked cabinet in the MC Phnom Penh office, and will be available during data cleaning and analysis. Surveys will be maintained in storage for a period of 2 years after completion of the study.

### Statistical methods

All statistics will be performed in R (Murray Hill, NJ, USA) and Stata® (College Station, TX, USA).

#### Primary outcome

Adult female *Aedes* density will be summed over follow-up time points to give a single rate per cluster. This will be analyzed by negative binomial regression using the number of adults as the response, and the logarithm of the sampling effort (that is, person-time spent aspirating) as an offset. Hence, this analysis will yield density ratios. The primary analysis will not be adjusted, but secondary analysis will include an analysis adjusted for the baseline density.

#### Secondary outcomes

Secondary outcomes including entomological indices, such as HI, CI, BI, PPH, and PPP and dengue viral infectivity rate, will also be analyzed by the above methods.

#### Missing data

Missing data will be reported as recommended by Díaz-Ordaz et al. [57] and their impact may be explored in secondary analyses.

### Data monitoring

In accordance with the findings of Grant et al., we have not established a Data Safety Monitoring Board for this study as it is not a “clinical trial evaluating a therapy with a mortality or irreversible morbidity endpoint” [58]. However, a Technical Steering Committee (TSC) was established which will meet at least every 6 months and address any safety or other concerns that may arise. The TSC will have one member from each of the partner organizations including the government and WHO. HC and CHW staff have been advised to contact MC staff should any adverse event be detected through passive monitoring as a result of project activities. CHW monthly monitoring forms will also record any adverse events (such as tingling in the hands after touching PPF or gastrointestinal effects after potential exposure of PPF to the mouth) that are reported. Any event will be promptly reported to the ethics committees and government partners. If an end to the trial is needed, the decision will come from the chair of the TSC. However, no harms are foreseen, and trials of similar products have not experienced any adverse events or unintended effects [16, 17].

### Access to data

All co-principal investigators and partners will be given access to the cleaned datasets without identifiers, which will be stored on the Malaria Consortium Sharepoint site and will be password protected. The final dataset will also be stored in the Cambodian National Center for Parasitology, Entomology, and Malaria Control central repository.

### Ancillary and post-trial care

In the event of any harm associated with the protocol MC will be responsible as the trial sponsor. The control group will be receiving routine interventions from CNM as described above and will continue to receive them after the close of the project. After the end of the project the lead institution, MC, will continue to advocate for, and encourage uptake of, any policy recommendations that come from the study.

### Dissemination policy

The principal investigator (Jeffrey Hii) will ensure that the results of the trial are published regardless of outcome. At least every 6 months results will be shared with the Technical Steering Committee. In addition to reporting the results in peer-reviewed journals, the results will disseminated at the provincial level and national level to all project stakeholders. All documents and study materials will be made available in a tool kit that will be given to all government stakeholders and partners. The investigators will also disseminate their findings in international scientific conferences. Reporting will follow the guidelines in the Consolidated Standards of Reporting Trials (CONSORT) Statement [59]. Authorship will follow MC authorship guidelines which require substantive contributions to the design, conduct, interpretation, and reporting of a trial. The full protocol, household-level dataset, and statistical code will placed in the Cambodian Ministry of Health’s central repository within 6 months of completion where all interested researchers can request access.

## Discussion

Due to the rise in dengue cases [3] and the current lack of effective vaccines and therapeutics, there is an urgent need to develop more effective vector-control methods [22]. These methods together with the development of new vaccines [12], genetic control of mosquitoes [14, 15], and new therapeutic drugs [60] will be essential in reducing dengue prevalence throughout the world. Additionally, evidence suggests that the main vector-control tool in Cambodia (temephos) is becoming less effective [19, 20], and a need to assess new sustainable vector-control methods in this context exists [22].

Recent studies have suggested the use of larvivorous fish to be effective in vector control [21, 22]; however, many were methodologically flawed and none have used a randomized controlled design [37]. The studies on previous products similar to Sumilarv® 2MR showed positive results [16, 17]; however, the new product has not been tested externally beyond small ongoing semi-field trials in Thailand (personal communication, Muney Serit, 2015). Evidence from larger trials is essential when trying to understand the true impact of these vector-control tools and in making recommendations to government bodies and donors.

The study area is suitable for the current trial as the disease is prevalent in the selected districts, and the province has a history of dengue outbreaks [20]. The study team is also familiar with the area having conducted multiple dengue research projects in the area, and have good relationships with the local authorities and communities in the area.

It would be preferable to have a primary outcome directly related to dengue incidence rather than an entomological one. Finding the appropriate metric to measure disease impact is bedeviled by the effect of human movement on patterns of transmission, and the pronounced temporal and spatial heterogeneity in transmission, which will necessitate very large cluster randomized study designs. We considered passive surveillance for dengue with rapid diagnostic tests in HCs. Although sensitivity among currently available tests was considered acceptable for routine clinical diagnostics [61] it was not considered high enough for seroconversion studies. No studies had used rapid diagnostics to estimate seroprevalence. Therefore, more expensive and labor-intensive efforts were preferable, such as cohort studies or capture-recapture methods (which have their own limitations [62]) to estimate the true number of cases, and using a more sensitive diagnostic tool such as RT-PCR. However, due to budget limitations it was not possible to employ them. Additionally, unpublished data from a recent cohort study in the proposed districts suggest that, given similar number of cases during this study timeframe, and the resources available to the current project, there would not be enough statistical power to show an impact of the likely size on case numbers. (personal communication, Agus Rachmat, 2015). Therefore, the endpoint chosen was the density of adult *Aedes* mosquitoes, which are on the causal pathway to disease.

There is always a need to balance potential benefits and harms during a trial. The potential benefits of the trial are substantial, and trials of similar interventions in the past have not experienced any adverse events or unintended effects [16, 17, 21, 22]. Additionally, because of the low acute toxicity of PPF it is considered extremely safe and is recommended by the WHO for use in drinking water [44].

This trial is designed to measure the reduction in adult and juvenile mosquitoes due to these vector-control methods relative to a control. However, one limitation is that the study was powered to detect a statistically significant difference between each arm compared with the control, and not between the intervention arms. This reduces the ability to see the impact of the PPF. A possible source of bias may be not having data collectors blind to the intervention; however, in this case it is unavoidable as data collection teams will be able to see the interventions in the containers which they sample. Contamination (spillover) of COMBI activities from intervention villages could affect our study by increasing knowledge or use of guppy fish in control areas. However, in the previous study it was found that only about 5% of containers had guppies in the control area at the end of the project [22]. Measurements of guppy fish coverage will also be conducted in control villages to identify the extent of any contamination.

Although these data are being collected within one province in Cambodia, it is likely that the result of this trial could be generalizable to areas with similar ecology within the country and in neighboring countries. Each country or province will have to make its own decision based on individual contexts. For example, unpublished MC studies in Myanmar showed similar size and types of containers and community practices in two regions, and interest from government officials in introducing guppies to water containers in response to dengue outbreaks (personal communication, Jeffrey Hii, 2015). However, the decision was made to not introduce guppies in the Philippines as the community acceptance was low and the cool climate in higher altitudes was not suitable for guppy survival and reproduction (personal communication, Jeffrey Hii, 2015).

### Trial status

At the time of submission of this manuscript the trial had completed the baseline data collections, enrollment of villages, and randomized allocation of the villages to three study arms.

## Additional files

Additional file 1: SPIRIT Checklist. (DOCX 42 kb)

Additional file 2: Study Consent Form. (DOCX 2385 kb)

## Abbreviations

BI: Breteau Index
*Bti*: *Bacillus thuringiensis israelensis*
CHW: Community health worker
CI: Container Index
COMBI: Communication for Behavior Impact
FGD: Focus Group Discussions
GIZ: Deutsche Gesellschaft für Internationale Zusammenarbeit
HC: Health center
HH: Household
HI: House Index
IDI: In-depth Interviews
KAP: Knowledge, Attitudes, and Practice
MC: Malaria Consortium
NAMRU-2: US Naval Medical Research Unit-2
NDCP: Cambodian National Dengue Control Program
OD: Operational district
PCI: Premise Condition Index
PPF: Pyriproxyfen
PPH: Pupae Per House
PPP: Pupae Per Person
TSC: Technical Steering Committee
