# Supplementary figures and images for: Determining the efficacy of guppies and pyriproxyfen (Sumilarv® 2MR) combined with community engagement on dengue vectors in Cambodia: study protocol for a randomized controlled trial
Source: Trials. 2017 Aug 4;18:367. doi: 10.1186/s13063-017-2105-2 (PMC5545006; doi:10.1186/s13063-017-2105-2)

Informed Consent Form
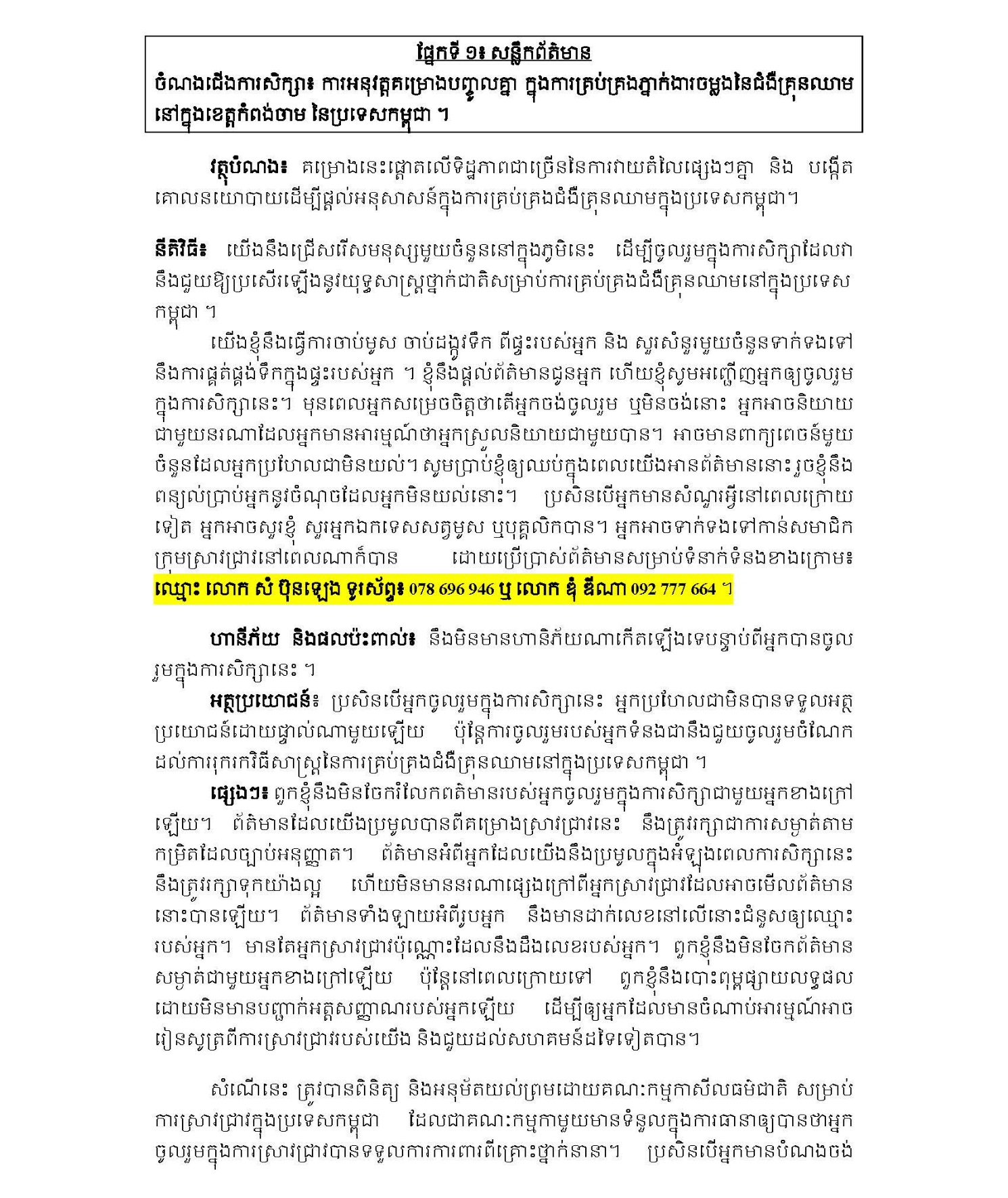


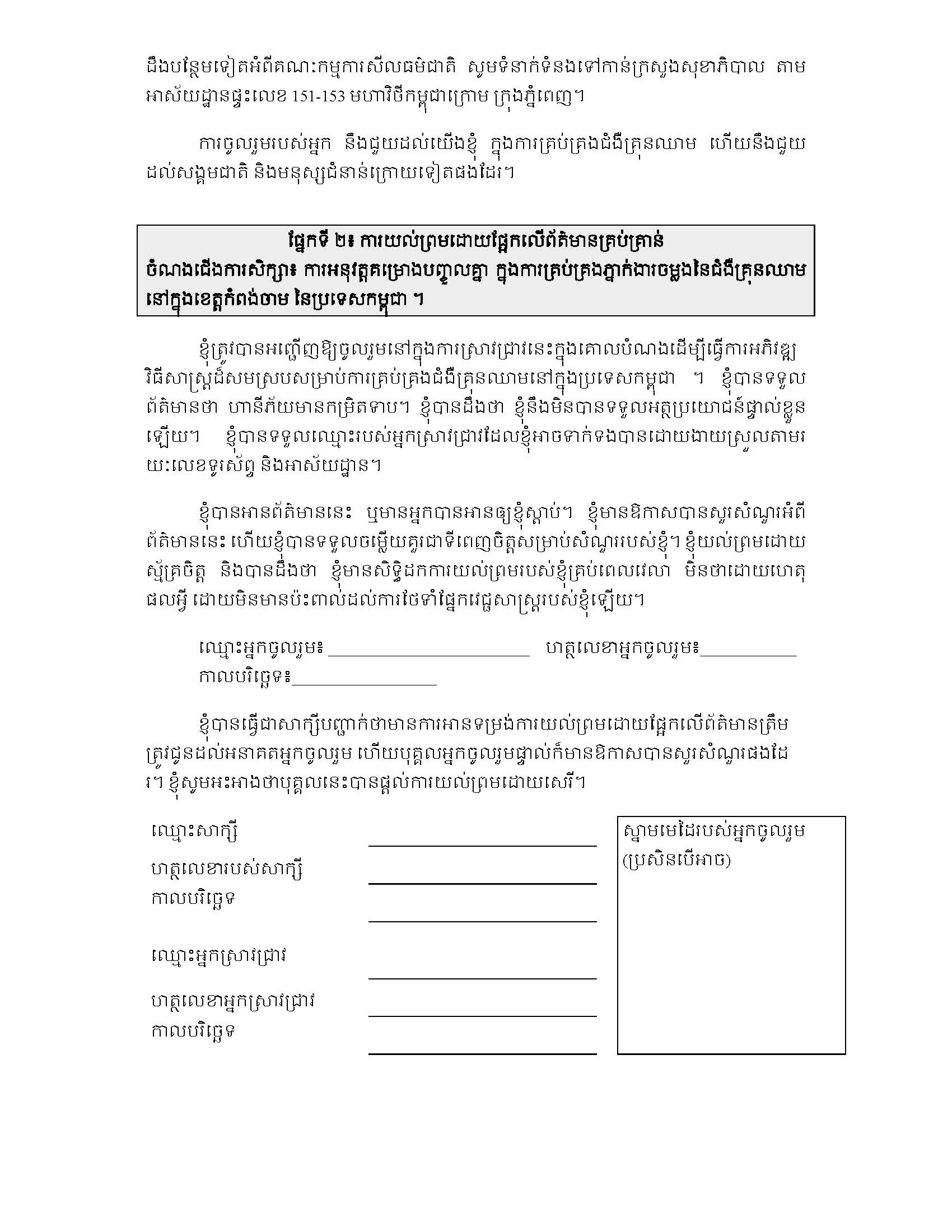

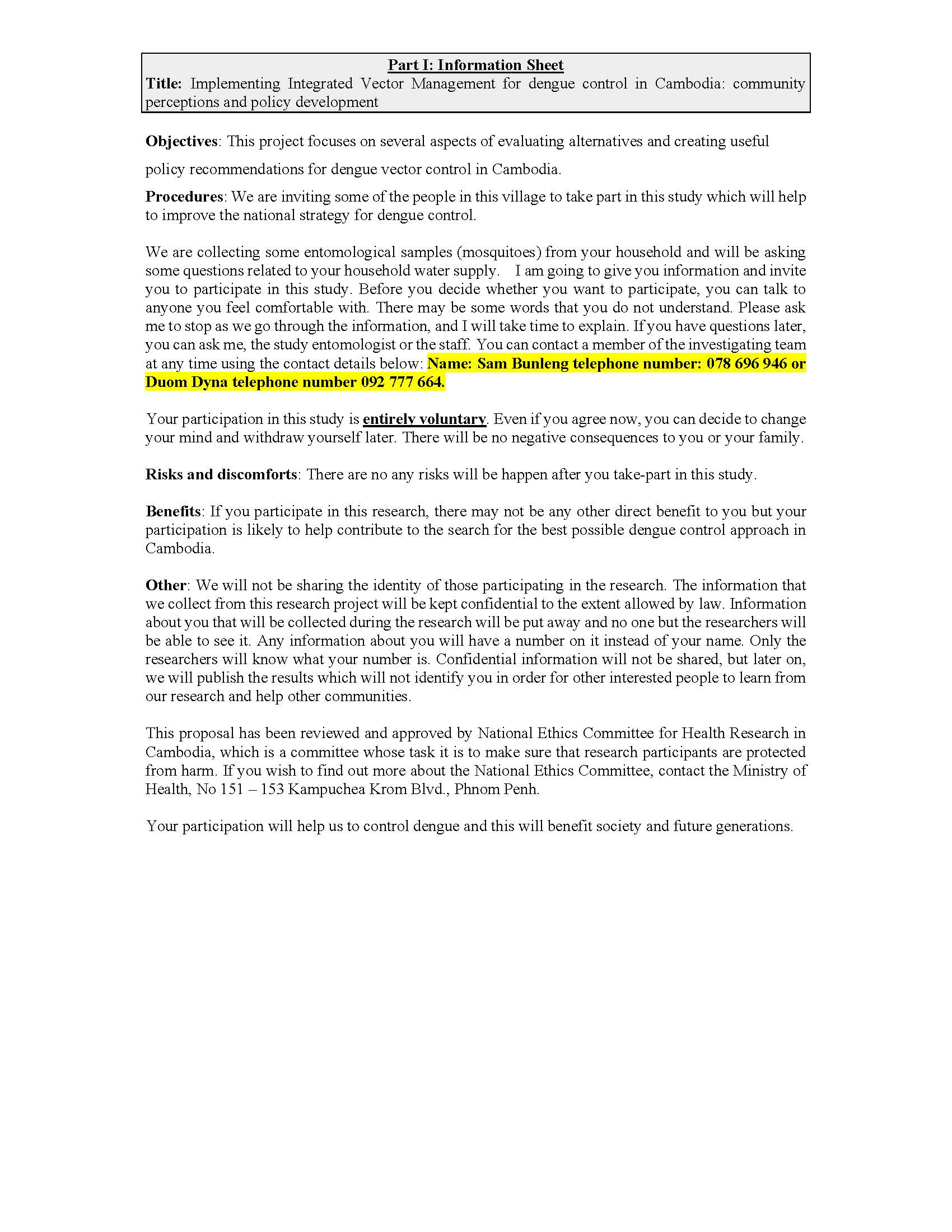

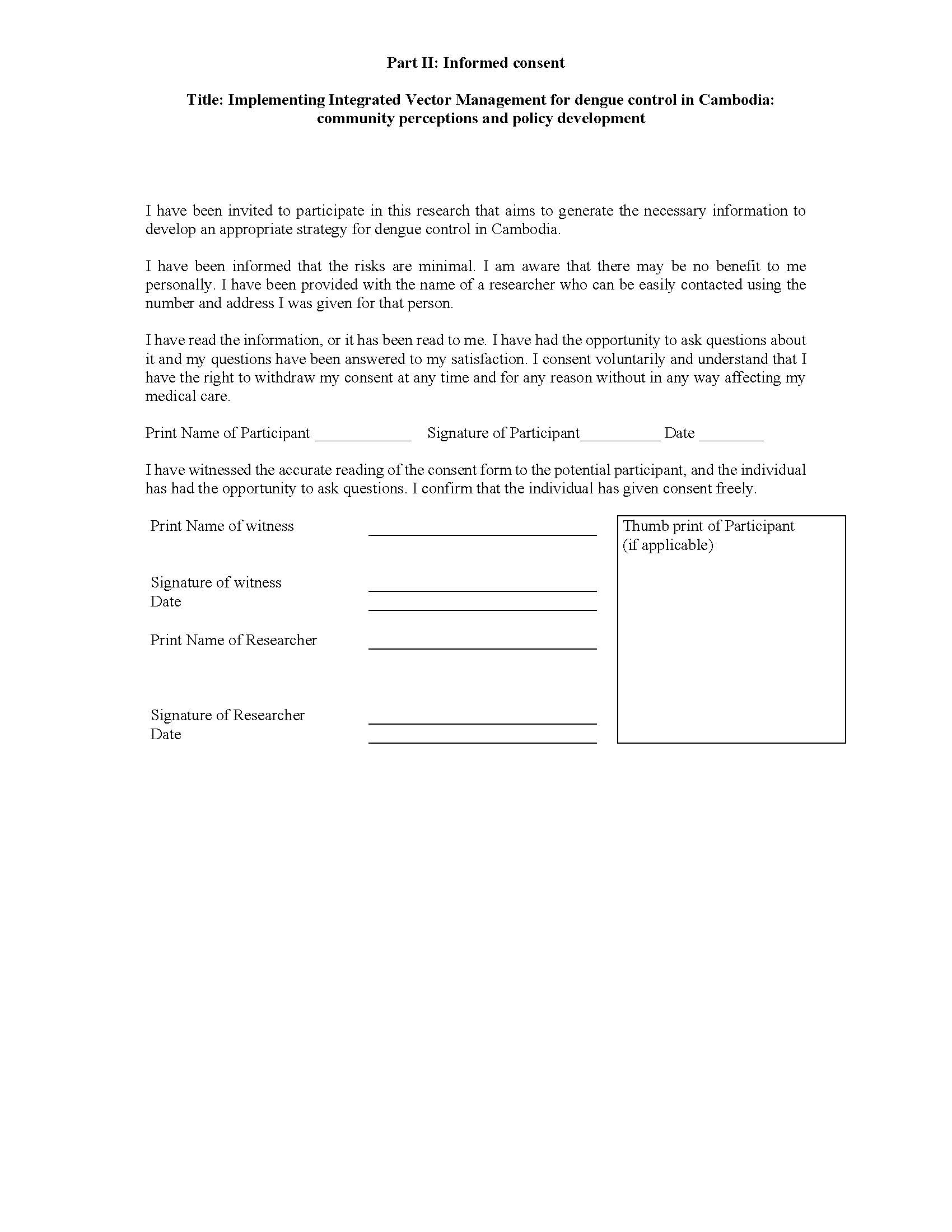


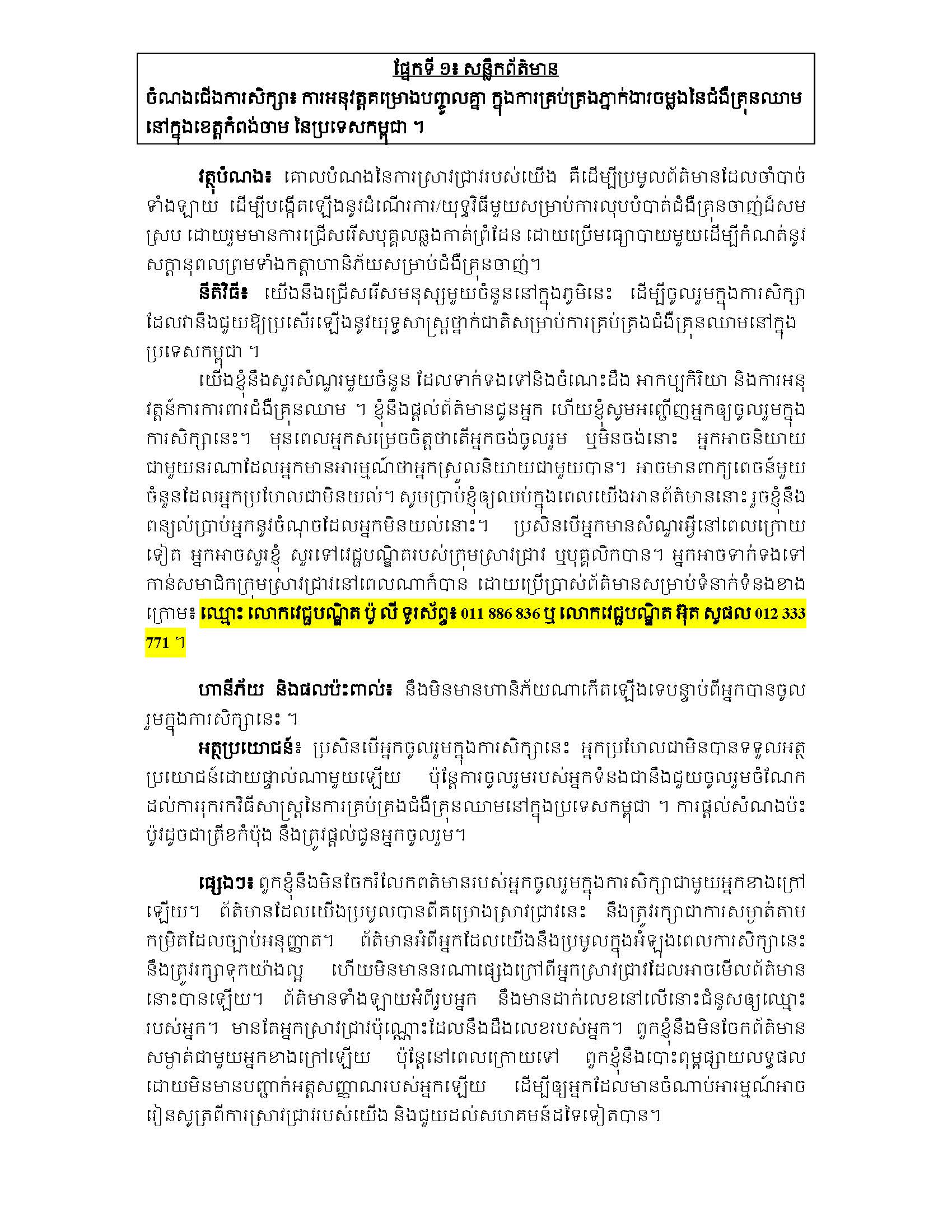


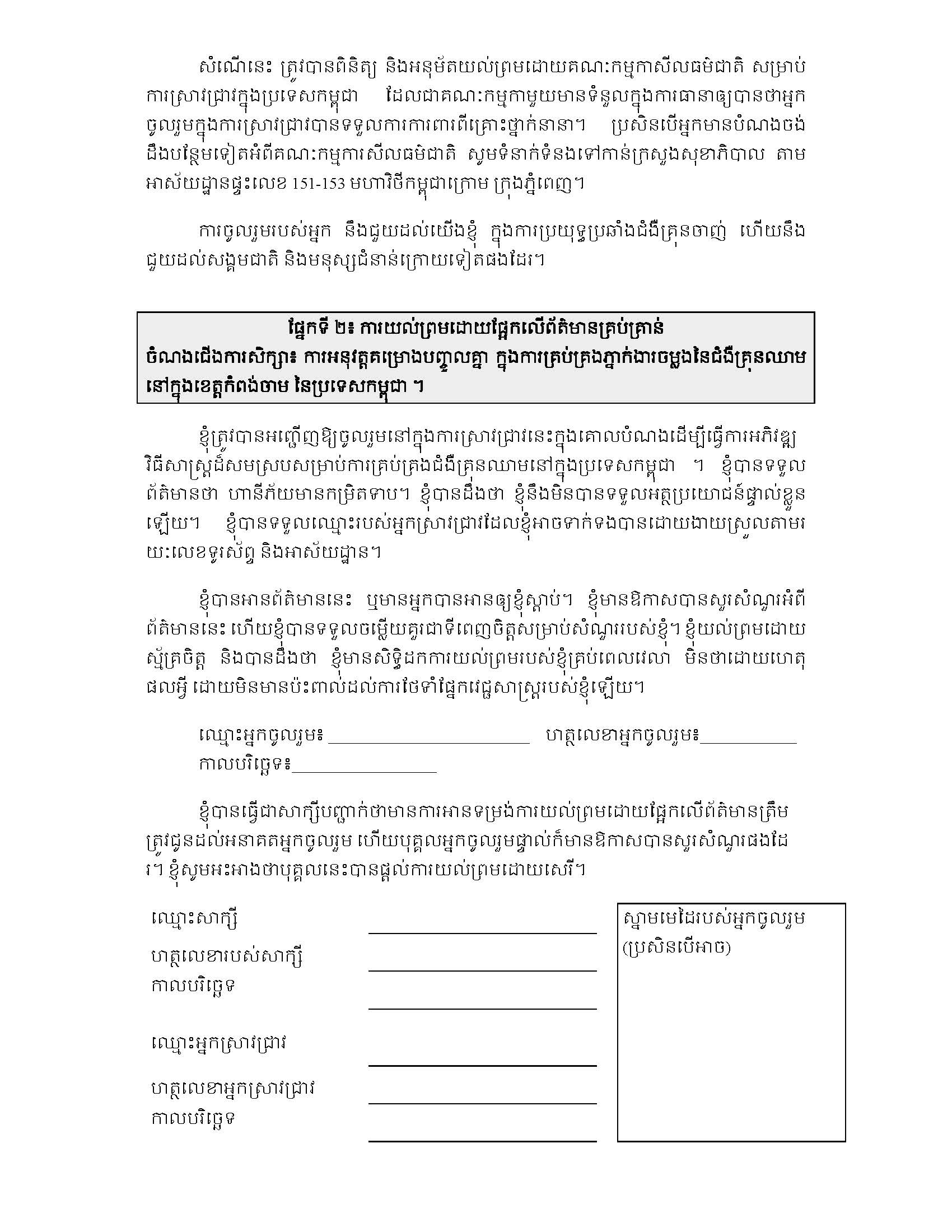

Supplement: Supplementary file 2 — Study Consent Form. (DOCX 2385 kb) [file 13063_2017_2105_MOESM2_ESM.docx]
